# *Arcobacter butzleri* Biofilms: Insights into the Genes Beneath Their Formation

**DOI:** 10.3390/microorganisms10071280

**Published:** 2022-06-23

**Authors:** Adrián Salazar-Sánchez, Itsaso Baztarrika, Rodrigo Alonso, Aurora Fernández-Astorga, Ilargi Martínez-Ballesteros, Irati Martinez-Malaxetxebarria

**Affiliations:** 1 MikroIker Research Group, Department of Immunology, Microbiology and Parasitology, Faculty of Pharmacy, University of the Basque Country UPV/EHU, Paseo de la Universidad 7, 01006 Vitoria-Gasteiz, Spain; adrian.salazar@ehu.eus (A.S.-S.); itsaso.baztarrica@ehu.eus (I.B.); rodrigo.alonso@ehu.eus (R.A.); aurora.fernandez@ehu.eus (A.F.-A.); ilargi.martinez@ehu.eus (I.M.-B.); 2 Bioaraba, Microbiology, Infectious Disease, Antimicrobial Agents, and Gene Therapy, 01006 Vitoria-Gasteiz, Spain

**Keywords:** *Arcobacter butzleri*, biofilm, knockout mutants, Congo red assay, static biofilm assays

## Abstract

*Arcobacter butzleri*, the most prevalent species of the genus, has the demonstrated ability to adhere to various surfaces through biofilm production. The biofilm formation capability has been related to the expression of certain genes, which have not been characterized in *A. butzleri*. In order to increase the knowledge of this foodborne pathogen, the aim of this study was to assess the role of six biofilm-associated genes in campylobacteria (*flaA*, *flaB*, *fliS*, *luxS*, *pta* and *spoT*) in the biofilm formation ability of *A. butzleri*. Knockout mutants were constructed from different foodborne isolates, and static biofilm assays were conducted on polystyrene (PS), reinforced glass and stainless steel. Additionally, motility and Congo red binding assays were performed. In general, mutants in *flaAB*, *fliS* and *luxS* showed a decrease in the biofilm production irrespective of the surface; mutants in *spoT* showed an increase on stainless steel, and mutants in *pta* and *spoT* showed a decrease on reinforced glass but an increase on PS. Our work sheds light on the biofilm-related pathogenesis of *A. butzleri*, although future studies are necessary to achieve a satisfactory objective.

## 1. Introduction

*Arcobacter butzleri* is a Gram-negative bacterium with a wide environmental distribution, classified as a foodborne pathogen [1] due to its association with human gastrointestinal disease. *A. butzleri* is the most prevalent among the species of the genus and is frequently isolated from wild and farm animals’ excrements and intestinal regions (boar, ostrich, Eurasian collared dove and raccoon), farm animals’ meat (chicken, pork, beef, turkey, lamb, sheep, rabbit and quail meat), seafood products (clam, mussel, cockle, squid and shrimp), dairy products (raw cow milk and fresh cheese), vegetables (carrot, spinach, lettuce, chard, parsley, arugula and radish), environmental water and human stool [2,3,4,5,6,7,8,9,10,11,12,13].

The transmission of the species of the genus *Arcobacter* through the food chain seems to be favored by their ability to form biofilms [14,15,16]. Many food-related pathogens such as *Aeromonas* spp., *Salmonella* Typhimurium, *Staphylococcus aureus*, *Listeria monocytogenes*, *L. ivanovii*, *Escherichia coli*, *Bacillus cereus*, *Cronobacter sakazakii*, *C. muytjensii*, *A. butzleri*, *A. cryaerophilus*, *Pseudomonas aeruginosa*, *Klebsiella pneumoniae*, *Campylobacter jejuni* and *C. coli* can produce these resistance structures conformed by cells and extracellular compounds [17,18,19,20,21,22,23,24,25]. The biofilm formation ability of *Arcobacter* species on different surfaces has also been documented [5,25], and it has become apparent that there is a notorious variability of the adherence level among strains of the same species. Due to the difficulty of eradicating biofilms and the rapidity with which *A. butzleri* develops them, preventing their formation is vital to control the spread of this foodborne pathogen [25]. Moreover, some authors referred that these structures could also contribute to the increase in antimicrobial resistance of *A. butzleri* since biofilm growth favors resistance gene transmission [24].

The biofilm formation process integrates different steps [26]. When planktonic bacteria find a stressful situation (i.e., carbohydrate, protein, fatty acid or other nutrient deficits; antibiotic exposure; and unfavorable temperature/atmosphere conditions [27,28,29]), cells start to adhere in a smooth and reversible manner to an available surface and to each other. Then, the production of exopolysaccharide (EPS) by the loosely adhered bacteria and/or the expression of specific adhesins located on pili and fimbriae leads to their irreversible attachment. At this point, sessile bacteria begin to form microcolonies, and the biofilm matures as they continue growing and producing EPS [30,31,32]. Finally, when the biofilm reaches a critical mass, cells detach from the outermost lawyer of growth, and the dispersion of planktonic cells from the biofilm occurs. The composition of the biofilm formed by *Arcobacter* still remains unknown, but, in general, the biofilm matrix is a complex structure that presents channels and pores throughout nutrients, oxygen and water flow. It is composed of approximately 15% of cells and 85% of EPS; this one is almost entirely conformed by water and contains in its’ solid-phase mainly polysaccharides, proteins and DNA [24,33].

Biofilm formation is dependent on many extrinsic factors such as growth medium, atmosphere, temperature, time, inoculum density and surface, but also on the intrinsic characteristics of each strain [14,15,25,34]. The biofilm formation capability has been related to the expression of genes of such diverse functions as motility, EPS production and cell signaling in many different bacteria. The expression of some of those genes is cell density-dependent, as the (p)ppGpp synthetase (i.e., *spoT*, *relA*, *relP*) and *Quorum Sensing* (QS) genes (i.e., *luxS*) [30]. In fact, low cell densities have been related to cell adhesion and high ones to cell detachment mediated by (p) ppGpp synthetases, and high cell densities also to biofilm formation as a result of QS autoinducer production [26,35,36]. In certain bacterial species, such as *E. coli*, *P. fluorescens*, *P. aeruginosa*, *B. subtilis*, *Agrobacterium tumefaciens* and *Shewanella oneidensis*, flagella and other surfaces structures such as fimbria, extracellular membrane proteins and amyloid-like fibrils are essential in the initial bacterial attachment and subsequent biofilm formation [24,26,30,37,38,39,40,41,42,43,44,45,46,47,48,49,50,51]. In fact, it has been demonstrated that functional flagella are necessary for maximum biofilm formation in species such as *Campylobacter jejuni*, *A. tumefaciens* and *E. coli*. This affirmation is supported by studies where the mutation of genes implicated in the synthesis of the flagellar filament (*flaA*, *flaB*, *flaG*, *fliA* and *fliS* in *C. jejuni*; and *fliC* in *E. coli*) [28,40,50,51,52] and hook (*flgE* in *A. tumefaciens* and *P. aeruginosa*) [46,53], flagellum movement (*motA* in *A. tumefaciens*, *C. jejuni* and *E. coli*) [44,46,50,52] and flagellar gene regulation (*fliA* and *qseB* in *E. coli*, and *fliW* in *C. jejuni*) [44,54] showed reduced biofilm formation or no formation at all. Further, higher expression levels of the *flaA* gene were reported for *L. monocytogenes* growing in biofilm compared to planktonic form [55].

The gene *spoT* encodes the bifunctional (p) ppGpp synthase/hydrolase SpoT [56]. Among others, this alarmone has been related to flagellar gene regulation and biofilm formation as part of the stringent response in many bacteria such as *E. coli*, *Legionella pneumophila*, *Mycobacterium tuberculosis*, *C. jejuni* and *Helicobacter pylori* [56,57,58,59]. Lower expression levels of the flagellar genes *flgH* and *flgE* were noticed in *spoT* mutant strains in *Vibrio* spp [26,60]. The lack of flagella hindered the initial attachment and delayed the biofilm formation [26,60]. In *H. pylori*, *spoT* mutants formed lighter biofilms than the wild type, showing differences in the matrix conformation [59]. This gene has also been directly related to the upregulation of biofilm formation in *E. coli*, *Streptococcus mutans*, *H. pylori* and *C. jejuni* [59,61,62].

In 2015, Kim et al. [63] noticed that phosphate acetate (AcP) could play a role as a mediator in the expression of genes such as *relP* (a short RelA/SpoT Homologue (RSH) with alarmone synthase function [27,64]) and *luxS* (implicated in QS). The AcP is created via the Pta-AcK pathway, which has also shown an implication in the biofilm formation process of different bacteria such as *S. mutants*, *E. coli* and *C. jejuni* [40,63,65]. This pathway is composed of the enzymes Pta (i) and Ack (ii), encoded by *pta* and *ackA*, respectively, that work (i) transforming the acetyl-CoA into AcP and (ii) AcP into acetate [63,66]. In a recent transcriptional study of *Campylobacter* spp., the presence of the *pta* gene was related to biofilm production and its absence to weak or no-biofilm formation [67]. In contrast, other studies related the absence of *pta* with a biofilm increase in different species [40,65,68]. On the other hand, the highly conserved *luxS* gene [69,70,71] encodes the LuxS metalloprotease. This enzyme is involved in the production of the autoinducer-2 (AI-2), one of the most studied QS signaling molecules [35]. In the biosynthesis of AI-2, S-adenosylhomocysteine (SAH) is hydrolyzed to S-ribose homocysteine (SRH) by the enzyme Pfs, then transformed into 4,5-dihydroxy-2,3-pentanedione (DPD) by LuxS and finally self-cycled to form the AI-2 [35,72,73]. The LuxS/AI-2 QS pathway is related to a variety of processes such as biofilm production, plasmid transference, motility, drug resistance, adhesion and virulence-gene expression [69,74,75,76,77]. In fact, *luxS* has been found necessary for an efficient biofilm formation in *S. mutans*, *V. cholerae*, *Salmonella* Typhi, *L. monocytogenes*, *Lactobacillus rhamnosus*, *H. pylori*, *C. jejuni* and *Porphyromonas gingivalis* [28,63,78,79,80,81,82,83].

*A. butzleri* presents homologous of the genes *flaA*, *flaB*, *fliS*, *luxS*, *pta* and *spoT* [5], but their function related to the biofilm formation has not been established yet. Nevertheless, it is reasonable to think that they may also affect adherence and biofilm formation in *A. butzleri*. Understanding the mechanism beneath the biofilm formation is vital for designing potential control strategies. Therefore, in order to increase the knowledge of *A. butzleri* pathogenesis, the aim of this study was to assess the role of six biofilm-associated genes in campylobacteria (*flaA*, *flaB*, *fliS*, *luxS*, *pta* and *spoT*) [28,40,62,67,84] in the biofilm formation ability of *A. butzleri*.

## 2. Materials and Methods

### 2.1. Bacterial Strains, Plasmids and Growth Conditions

Four *A. butzleri* strains were selected for mutagenesis assays of biofilm-associated genes based on their different ability to form biofilms [5,14]. All of them had been previously isolated from different food products at retail [2,5] and presented biofilm-associated genes, as confirmed by PCR (see the following section). The reference strain *A. butzleri* RM 4018 was also included. All the strains and plasmids used in this study are listed in Table 1.

*Arcobacter* strains were routinely grown at 37 °C in Brain Hearth Infusion (BHI) broth (Oxoid, Basingstoke, UK) or on Columbia agar base plates (Oxoid, Basingstoke, UK) supplemented with 5% defibrinated horse blood (Liofilchem, Roseto degli Abruzzi, Italy). *Escherichia coli* strains were routinely grown at 37 °C in Luria-Bertani (LB) broth or on LB agar plates (Condalab, Torrejón de Ardoz, Spain), supplemented with ampicillin (100 µg/mL) (CAS: 69-52-3; Sigma-Aldrich, St. Louis, MO, USA) or kanamycin (50 µg/mL) (CAS: 25389-94-0; NZYTech, Lisbon, Portugal) when necessary. In both cases, the media were incubated aerobically for 24–48 h.

### 2.2. Growth Curve Measurements

Overnight liquid cultures were diluted into fresh BHI to reach an optical density of 0.05 at 550 nm (OD_550_). Two hundred microliters of each prepared bacterial suspension were inoculated into four wells of a 96-well flat-bottom microtiter plate (Nest Biotechnology, Wuxi, China), and the plates were placed in a Synergy^TM^ HT plate reader (BioTek, Winooski, VT, USA) to monitor the OD_550_ of the bacteria every hour for 24 h. The plates were maintained under aerobic conditions at 37 °C and agitated at 17 Hz. The exponential growth rate was calculated from three independent growth experiments.

### 2.3. Biofilm-Associated Gene Detection

The presence of six biofilm-associated genes *(flaA*, *flaB*, *fliS*, *luxS*, *pta* and *spoT*) was determined by individual PCRs performed on 100 ng of DNA with 1.25 U of Supreme NZYTaq II DNA Polymerase (NZYTech, Lisbon, Portugal), 0.1 mM of each dNTP, 1X buffer and 0.25 µM of each primer set. All the primers used in this study are listed in Supplementary Materials Table S1. The PCR parameters were 5 min at 95 °C; 30 cycles of 94 °C for 30 s, annealing temperatures ranging from 50 to 56 °C for 30 s and 72 °C for 1 min; and 10 min at 72 °C. DNA from *A. butzleri* RM4018 was used as the positive control and deionized water as the negative one.

### 2.4. Construction of Knockout Mutant Strains

Knockout (KO) mutants for biofilm-associated genes were constructed according to a previously described method [85] with some modifications. Briefly, the genes and their flanking regions were amplified by PCR using the proofreading enzyme ACCUZYME^TM^ DNA polymerase (Bioline, Memphis, TN, USA), 5′-A tailed using BIOTAQ^TM^ DNA polymerase (Bioline, Memphis, TN, USA) and cloned in the commercial cloning vector pGEM-T Easy (Promega, Madison, WI, USA). The resulting plasmids were linearized either by an outward PCR performed with *Bam*HI cutting site containing primers (pGflaAB) or by restriction enzyme digestion with *Mun*I, *Cla*I, *Bmt*I or *Afl*II (pGfliS, pGluxS, pGpta and pGspoT, respectively). The linearized plasmids were ligated to a kanamycin (Km) resistance cassette (*aph(3*′*)-III)* obtained from the pMW2 plasmid [86] either by *Bam*HI digestion or by PCR amplification using primers that contained the appropriate restriction cutting site for each case. The orientation of the cassette and the ORFs was the same in all the constructed plasmids.

### 2.5. Motility Assays

The motility of the strains was assayed by stab-inoculation of single colonies into thioglycolate semisolid agar plates (thioglycolate medium containing 0.4% agar) (Scharlau, Sentmenat, Spain). The plates were incubated under aerobic conditions at 37 °C for 24 h, and the diameter of the motility zone was measured. The assays were carried out at least on three independent occasions.

### 2.6. Biofilm Formation Assays

The biofilm formation ability of the strains on polystyrene (PS), reinforced glass and stainless steel was evaluated following previously described protocols [14] with minor modifications. For PS and reinforced glass assays, the inocula were adjusted to an optical density of 0.2 at 600 nm (OD_600_), the incubations were performed at 37 °C for 48 h, and the biofilms were stained with 200 µL of crystal violet (1% water solution) (CAS: 548-62-9; Sigma-Aldrich, Steinheim, Germany). For stainless-steel assays, 7 mL of an OD_600_ = 0.2 cell suspension was added to each coupon-containing tube, and the incubations were performed at 37 °C for 24 h. After gently washing with distilled water, the coupons were transferred to 15 mL conical plastic tubes containing 7 mL of sterile 0.01 M phosphate-buffered saline (PBS) and 15 glass pearls. The biofilms formed on PS and reinforced glass were expressed by the biofilm formation index (BFI) according to Niu and Gilbert (2004) [87], and the strains were subsequently categorized as strong, moderate, weak or non-biofilm formers according to Naves et al. (2008) [88]. The biofilms formed on stainless steel were evaluated by plate count method on Mueller–Hinton agar (Oxoid, Basingstoke, UK), expressing the results as logUFC/cm^2^.

### 2.7. Congo Red Binding Assay

For each strain, isolated colonies from overnight cultures were suspended in physiological saline (0.9% (*w*/*v*) NaCl) to 0.5 McFarland. Three 10 µL drops of each inoculum were added onto Congo Red Agar (CRA) plates, which were composed of 37 g/L Brain Hearth Infusion broth (Oxoid, Basingstoke, UK) and 10 g/L of Bacteriological Agar (Scharlau, Sentmenat, Spain), supplemented with an autoclave-sterilized concentrated Congo red (CAS: 573-58-0; Acros Organics, Geel, Belgium) solution (20 mg/mL) in a final concentration of 0.1 g/L [89,90,91]. After 48 h of incubation at 37 °C, the CRA plates were observed against a backlight to differentiate the color of the colonies. Four strains were tested on each plate, and the experiments were performed a minimum of three independent times. Strains with a red phenotype on CRA plates were considered cellulose producers and those with a white one as non-producers.

### 2.8. Data Analysis

Statistical analyses were performed using the IBM SPSS Statistics 26 software (IBM Corp., New York, NY, USA). The normality of the numerical values obtained for each strain was determined by the Shapiro–Wilk test. The adhesion capacity, growth rate and motility of the strains were compared by Student’s *t*-test and one-way analysis of variance (ANOVA). Significant differences were established at *p* values of <0.05.

## 3. Results

### 3.1. Construction of Knockout (KO) Mutants and Growth Analysis

Overall, all the genes were successfully knocked out. A total of 18 KO mutant strains were obtained from the five *A. butzleri* studied strains (Table 1). All the five expected mutants were obtained from the strains CCUG 30485 and CH11, four from CZ6, three from P8 and one from BER7. The most successfully mutated gene was *pta*, which could be inactivated in all the studied *A. butzleri* strains, followed by *fliS* and *luxS* in four. *spoT* and *flaAB* were inactivated in three and two strains, respectively. The comparison of the various mutants with their correspondent parent strain showed no differences in bacterial shape, colony formation on blood agar plates or growth rate in BHI (ANOVA-based *p* > 0.05) (Figure S1).

**Table 1:** Bacterial strains and plasmids used in this study.

Bacterial Strain or Plasmid	Source/Function	Reference	Biofilm Formation ^3^	Biofilm Associated Genes Detected by PCR
***Arcobacter butzleri* strains**				
BER7	Wild strain isolated from cockle	[5]	2.48 ± 1.16	*flaA*, *flaB*, *fliS*, *luxS*, *pta* and *spoT*
BER7Δ*pta*::Km	AB-BER7 derivative Δ*ABU_RS02465*::*aph(3′)-III*	This study		
CCUG 30485	Human clinical isolate (ATCC 49616; RM4018)	CCUG ^1^		*flaA*, *flaB*, *fliS*, *luxS*, *pta* and *spoT*
CCUG 30485Δ*flaAB*::Km	CCUG 30485 derivative Δ*ABU_RS11245- RS11250*::*aph(3′)-III*	This study		
CCUG 30485Δ*fliS*::Km	CCUG 30485 derivative Δ*ABU_RS01060*::*aph(3′)-III*	This study		
CCUG 30485Δ*luxS*::Km	CCUG 30485 derivative Δ*ABU_RS00560*::*aph(3′)-III*	This study		
CCUG 30485Δ*pta*::Km	AB-CCUG 30485 derivative Δ*ABU_RS02465*::*aph(3′)-III*	This study		
CCUG 30485Δ*spoT*::Km	AB-CCUG 30485 derivative Δ*ABU_RS03230*::*aph(3′)-III*	This study		
CH11	Wild strain isolated from squid	[5]	0.76 ± 0.13	*flaA*, *flaB*, *fliS*, *luxS*, *pta* and *spoT*
CH11Δ*flaAB*::Km	AB-CH11 derivative Δ*ABU_RS11245-RS11250*::*aph(3′)-III*	This study		
CH11Δ*fliS*::Km	AB-CH11 derivative Δ*ABU_RS01060*::*aph(3′)-III*	This study		
CH11Δ*luxS*::Km	AB-CH11 derivative Δ*ABU_RS00560*::*aph(3′)-III*	This study		
CH11Δ*pta*::Km	AB-CH11 derivative Δ*ABU_RS02465*::*aph(3′)-III*	This study		
CH11Δ*spoT*::Km	AB-CH11 derivative Δ*ABU_RS03230*::*aph(3′)-III*	This study		
CZ6	Wild strain isolated from quail	[5]	3.00 ± 2.90	*fliS*, *luxS*, *pta* and *spoT*
CZ6Δ*fliS*::Km	AB-CZ6 derivative Δ*ABU_RS01060*::*aph(3′)-III*	This study		
CZ6Δ*luxS*::Km	AB-CZ6 derivative Δ*ABU_RS00560*::*aph(3′)-III*	This study		
CZ6Δ*pta*::Km	AB-CZ6 derivative *ΔABU_RS02465*::*aph(3′)-III*	This study		
CZ6Δ*spoT*::Km	AB-CZ6 derivative Δ*ABU_RS03230*::*aph(3′)-III*	This study		
P8	Wild strain isolated from chicken	[2]	9.44 ± 6.07	*fliS*, *luxS*, *pta* and *spoT*
P8Δ*fliS*::Km	AB-P8 derivative Δ*ABU_RS01060*::*aph(3′)-III*	This study		
P8Δ*luxS*::Km	AB-P8 derivative Δ*ABU_RS00560*::*aph(3′)-III*	This study		
P8Δ*pta*::Km	AB-P8 derivative Δ*ABU_RS02465*::*aph(3′)-III*	This study		
***Escherichia coli* strains**				
*E. coli* DH5α NCCB2955	Competent cells for cloning	NCCB ^2^		
**Plasmids**				
pGEM-T Easy	Cloning vector, Amp^r^	Promega		
pGflaAB	pGEM-T Easy containing *ABU_RS11245-ABU_RS11250*	This study		
pGfliS	pGEM-T Easy containing *ABU_RS01060*	This study		
pGluxS	pGEM-T Easy containing *ABU_RS00560*	This study		
pGpta	pGEM-T Easy containing *ABU_RS02465*	This study		
pGspoT	pGEM-T Easy containing *ABU_RS03230*	This study		
pGΔflaAB	pGEM-T Easy containing Δ*ABU_RS11245-RS11250*	This study		
pGΔfliS	pGEM-T Easy containing Δ*ABU_RS01060*	This study		
pGΔluxS	pGEM-T Easy containing Δ*ABU_RS00560*	This study		
pGΔpta	pGEM-T Easy containing Δ*ABU_RS02465*	This study		
pGΔspoT	pGEM-T Easy containing Δ*ABU_RS03230*	This study		
pGΔflaAB::Km	pGEM-T Easy containing Δ*ABU_RS11245-RS11250*::*aph(3′)-III*	This study		
pGΔfliS::Km	pGEM-T Easy containing Δ*ABU_RS01060*::*aph(3′)-III*	This study		
pGΔluxS::Km	pGEM-T Easy containing Δ*ABU_RS00560*::*aph(3′)-III*	This study		
pGΔpta::Km	pGEM-T Easy containing Δ*ABU_RS02465*::*aph(3′)-III*	This study		
pGΔspoT::Km	pGEM-T Easy containing Δ*ABU_RS03230*::*aph(3′)-III*	This study		
pMW2	pBluescript KS M13 +:KmR (pILL550)	[86]		

^1^ CCUG: Culture Collection University of Gothenburg. ^2^ NCCB: Netherlands Culture Collection of Bacteria. ^3^ Data obtained from Martinez-Malaxetxebarria et al. [5] and Girbau et al. [14]. Values are expressed as mean ± standard errors.

### 3.2. Motility Assays

The motility of the strains, expressed in numerical values, is shown in Table 2. Representative images can be consulted in Figure S2. The five parent strains and most of the mutants (11 out of 18) were motile. In contrast, all those mutants in the flagellar genes (*flaAB* and *fliS*) and one in *pta* (BER 7 derivative) were non-motile. This loss of motility resulted as significant in all cases (*p* ≤ 0.001). Among the motile strains, all the obtained *spoT* and *luxS* mutants except P8Δ*luxS*::Km showed higher motility than their corresponding parent strains, and so did the CCUG 30485 and CH11-derivative mutants in *pta*. In contrast, CZ6 and P8-derivative mutants in the same gene showed lower motility than their parent strains. None of the observed differences were statistically significant.

### 3.3. Biofilm Formation Assays

The ability shown by the strains to form biofilms on different surfaces is resumed in Table 2. Under the experimental conditions, the majority of the strains (19 out of 22) formed biofilms on PS and were categorized as strong biofilm producers, especially P8 (*p* < 0.04). The exceptions were the strains CZ6, CZ6Δ*luxS*::Km and *CZ6ΔfliS*::Km, which did not show any adherence ability on this surface. Almost all mutants showed different biofilm formation abilities from their parent strains but the only significantly different one (*p* = 0.023) was that shown by BER7Δ*pta*::Km, which showed a BFI almost five times higher than BER7. On reinforced glass, all the strains formed biofilms, but their categorization differed from that on PS. Among the wild strains, BER7 was defined as weakly adherent, CZ6 as moderately adherent and CCUG 30485, CH11 and P8 as strongly adherent. On this material, all the mutant strains showed differences in their BFI values from their correspondent parent, and the ANOVA showed a significant reduction in the biofilms formed by CZ6Δ*fliS*::Km and P8Δ*fliS*::Km (*p* = 0.033 and 0.001, respectively). In general, the biofilm formation ability of the strains was higher on PS than in reinforced glass, and according to Student’s *t*-test, it was significant for CH11 (*p* = 0.019). Regarding stainless steel, viable cells could be recovered from all the coupons, which indicates the capability of all the studied strains to form biofilms on this surface. Based on the ANOVA, the adhesion of CZ6Δ*spoT*::Km was significantly higher than that of its parental on this material. (*p* = 0.006).

### 3.4. Congo Red Agar Assays

The wild BER7, CH11 and P8 strains turned out to be non-cellulose producers based on their phenotype on CRA plates (white growth). In contrast, the strains CCUG 30485 and CZ6 were cellulose producers (red growth). No differences were observed between wild and KO mutant strains. The pigmentation acquired by the strains when grown on CRA plates can be consulted in Figure S3.

## 4. Discussion

The transmission and pathogenicity of many bacteria are related to their capacity to form biofilms [14,15,16]. These structures have gained great interest over the last years, and the mechanisms underlying their formation and maintenance are being elucidated in many bacterial species [25,26,27,28,29,30,35,40,63,68,92]. Nevertheless, this knowledge is still scarce for the foodborne pathogen *Arcobacter butzleri*. To address this item, in this study, we aimed to understand the role of *flaA*, *flaB*, *fliS*, *luxS*, *pta* and *spoT* in the biofilm formation process of this species. For this purpose, mutants in the abovementioned genes that are associated with biofilm formation in other campylobacteria were constructed, and their biofilm formation ability on various surfaces of different hydrophobicity was compared to that of their parent strains. In addition, the capability to form biofilms was also tested using the Congo red binding assay, widely used by other authors in species such as *E. coli*, *K. pneumoniae*, *S. enterica* and *C. jejuni* [29,93,94].

Congo red indicator binds to curli/fimbria and cellulose [95,96] and considering that *A. butzleri* does not have curli/fimbria, the assay indicates cellulose production in this species. Our results did not show differences between mutant and parent strains, suggesting that the inactivated genes apparently do not take part in cellulose production in *A. butzleri*. As far as we know, this is the first time where cellulose production by *Arcobacter* has been reported. Moreover, we are not aware of the presence of genes involved in this process in *A. butzleri*, which leaves the way open for new research lines. On the other hand, although it has been satisfactorily used to detect biofilm production in campylobacteria [29], the results obtained on the Congo red binding assay did not correlate with those obtained on the biofilm formation assays in our case. Both cellulose-producing (red growth) and non-producing (white growth) strains formed biofilms under the experimental conditions. This phenomenon has been previously described in some other species [97]. Consequently, we do not consider the Congo red binding assay to be reliable for the identification of biofilm-forming bacteria in this species.

In accordance with previous studies held with both Gram-positive and negative bacteria [28,40,98,99,100], the results of this study point to the flagellum as an important structure implied in the biofilm formation of *A. butzleri*. The obtained mutants in the *flaA* and *flaB* genes (CCUG 30485Δ*flaAB*::Km and CH11Δ*flaAB*::Km) showed reduced biofilm-formation abilities compared with their parent strains in all the surfaces tested. Similarly, mutants of the *fliS* gene, which encodes the FliS chaperone responsible for flagellin protection and transport, adhered less than wild-type strains to PS and reinforced glass, especially CZ6Δ*fliS*::Km and P8Δ*fliS*::Km to the latter material (*p* = 0.033 and *p* = 0.001, respectively). However, three out of the four obtained *fliS* mutants, namely CCUG 30485Δ*fliS*::Km, CH11Δ*fliS*::Km and CZ6Δ*fliS*::Km, showed enhanced adhesion on stainless steel. The importance of a functional flagellum for maximum biofilm formation by campylobacteria has been previously reported. Studies held with *Campylobacter* spp. demonstrated that mutants on the *flaA*, *flaB*, *fliA*, *flaG* and *motA* genes showed reduced adhesion and biofilm formation ability [28,50,51]. Joshua et al. [40] observed that aflagellated *C. jejuni fliS* mutants were unable to attach to surfaces, and Hathroubi et al. [101] that aflagellated *H. pylori* mutants produced weaker biofilms. In other species, such as *V. cholerae* and *P. aeruginosa*, mutants with affectation in flagella showed compromised biofilm formation [102,103]. Despite not having tested the integrity of the flagella in our mutants in the *flaA*, *flaB* and *fliS* flagellar genes, the decreased motility observed for all of them could be indicative of their non-correct functionality. These three genes are essential for the synthesis and transport of the flagellin subunits that conform to the flagella filament [24,26,30,37,38,39,40,44,45,46,47,48]. Likely, the inactivation of any of these genes led to the production of lower flagellin levels and, consequently, to abnormal flagella with shorter filament or no filament at all, as previously reported elsewhere [104]. Mostly, the inactivation of flagellar genes is associated with a decrease in the ability to adhere [28,40,50,51,54,67,98,99,100,101,104]. In consonance with this, different transcriptional studies indicated that the expression of some flagellar genes is higher when bacteria grow on a biofilm compared to the planktonic state. *L. monocytogenes* seems to overexpress *flaA* when growing on biofilm [55]. Among campylobacteria, strongly adherent *Campylobacter* strains show higher expression levels of *flaB* and *fliS* than weakly adherent ones [67], and biofilm growing *H. pylori* upregulates various genes related to the formation of the flagellar apparatus [101]. Being primarily a mobility structure, the flagellum has important functions for biofilm formation as mechanosensing of surfaces [105,106] or being a component of the biofilm matrix [101]. Even so, and in line with previous observations [107,108,109], the enhanced adhesion observed in some of our flagellar mutants indicates that, though important, a functional flagellum is not essential for biofilm formation in *A. butzleri*. Future studies may elucidate whether a truncated filament (or its absence) has an influence on the composition or structure of the biofilm matrix. In addition, our results point to the possible presence of other surface-induced mechanisms involved in the early steps of the biofilm formation processes, as could be the adhesins CadF, PEB1a, JlpA, AcpA and CjaA, present in phylogenetically closely related species [110].

In general, the biofilms formed by *luxS* mutants were equal to or lower than those formed by the wild-type strains on the three studied surfaces. The role of the bacterial autoinducer-2 (AI-2) produced by *luxS* has been related to *Quorum Sensing* (QS), and this one with biofilm production [111]. The implication of QS in the biofilm production has been evidenced by the inactivation of genes coding for different signaling molecules and the subsequent reduction of the biofilm formed, such as the gene *lasI* in *P. aeruginosa* [112] and *cep* in *Burkholderia cepacia* [113]. The gene *luxS* has been found necessary for an efficient biofilm formation in *S. mutans*, *V. cholerae*, *Salmonella* Typhi and *P. gingivalis* [63,78]. In *S. mutans*, *K. pneumoniae* and *C. jejuni*, the lack of a functional *luxS* led to a decreased biofilm production [28,67,81,114,115,116], which is quite in accordance with our results. All our mutants in *luxS* adhered less than their parent strains except P8 and CZ6 derivatives on reinforced glass and stainless steel, respectively. Likewise, the absence of differences between the biofilms produced by wild-type and some *luxS* mutant strains (CZ6Δl*uxS*::Km in PS and all *luxS* mutants on stainless steel except CH11Δl*uxS*::Km) reported here had also been previously observed in *K. pneumoniae* [36] and *S. gordonii* [117]. In contrast, the inactivation of *luxS* increased the biofilm production in *H. pylori* [81,116]. Changes in biofilm morphology [69,114,117], motility reduction [52], decreased autoaggregation (which contributes to biofilm formation) [52], minimized growth [35] and reduced adhesion to cell lines [118,119,120] have also been reported due to the inactivation of *luxS*. Nevertheless, our results did not reflect growth differences between parent and *luxS* mutant strains.

Broadly, mutants of *pta* showed an increase in their biofilm formation ability on PS, which was statistically significant for BER7Δ*pta*::Km (*p* = 0.023), but a reduction on reinforced glass. The effect of this mutation in biofilm formation on stainless steel varied among the tested strains. The lack of *pta* has been associated with both increased and decreased biofilm formation. The inactivation of *pta* led to hyperflagellated *E. coli* mutant strains in a study conducted in 2005 [121]. According to the authors’ observations, the increased intracellular phosphate acetate (AcP) pool underlies the flagellar expression change. As mentioned above, flagella play an important role in biofilm formation; therefore, mutants of *pta* could show enhanced biofilm production if they overexpress flagellar genes. This could be the case with our *pta* mutants, which showed a general increase in motility and formed higher biofilms on PS than their correspondent parent strains. Increased biofilm productions derived from the inactivation of *pta* have also been reported in *E. coli*, *C. jejuni* and *S. mutans* [40,63,65]. Nevertheless, and based on the reported effect of high AcP levels in the expression of *luxS* and the RelA/SpoT system [63], we could hypothesize that the inactivation of *pta* and the subsequent AcP increase can lead to reduced concentrations of AI-2 and (p)ppGpp, synthesized by LuxS and RelA/SpoT systems, respectively; and, consequently, contribute to the decrease in flagella formation, EPS production and biofilm generation [26,27,35,57,58,60,61,63,64,71]. This would be in accordance with the biofilm formation reduction we report here for all our *pta* mutant strains on reinforced glass and for those derived from CZ6 and P8 on stainless steel, in line with the biofilm formation reduction reported in *S. mutans* [63].

Regarding the *spoT* mutants, when compared to their parent strains, all presented an increased ability to produce biofilms on PS and stainless steel and a reduced one on reinforced glass. This is in agreement with the reduced biofilm formation on glass reported for *V. cholerae* [61] and *Xanthomonas campestris* pv. *Campestris* 8004 [122] mutants. Similarly, enhanced biofilms on PS were noticed in *P. putida* KT2440 [123] and *V. alginolyticus* [26]. This gene, which encodes the bifunctional (p)ppGpp synthase/hydrolase, has been associated with obtaining maximum protection against stress (nutrient starvation, heat shocks, presence of NaCl and/or ethanol, etc.) and, therefore, facilitating bacterial pathogeny and dissemination [124,125]. Moreover, some authors have related it with the expression of various EPS operons (i.e., *vps*, *pea*, *peb*, *bcs*) in diverse bacterial genera, such as *Vibrio*, *Xanthomonas* and *Pseudomonas*; and, in consequence, with biofilm matrix production [26,61,122,123]. Moreover, the lack of *spoT* has also been related to low bacterial growth and no flagella formation, and, consequently, a reduced ability to form biofilms on glass tubes [61,126], which is consistent with our results on reinforced glass.

It is well known that extrinsic factors such as temperature, nutrient availability, surface material and environmental conditions influence biofilm formation in *Arcobacter* [14,15]. The methodology employed (growth media, incubation atmosphere and time, static/shaking culture, etc.) also affects it [111] and, therefore, contributes to the variability of results between studies. The differences between the BFI values obtained for the wild-type strains in this study (37 °C, BHI) and previous ones (30 °C, Arcobacter Broth) [5,14] are a good example of the influence the temperature and growth medium can have on bacterial adherence. Likewise, and in line with some previous studies [14,127,128], our results once again show that the hydrophobicity of the different surfaces (i.e., PS is hydrophobic and reinforced glass hydrophilic) has an effect on biofilm formation. Regardless of the mutated gene, PS seems to favor the adherence of *A. butzleri* under the experimental conditions, as almost all the strains were categorized as strong biofilm producers on this material. Nevertheless, an enhanced adhesion was previously reported for *A. butzleri* on glass [5], which again remarks on the great influence of the extrinsic factors on the process of biofilm formation.

In addition to providing useful information for the understanding of the biofilm-forming capability of *A. butzleri*, this first attempt to characterize the mechanisms involved opens up different research lines to gain additional insight into the biofilm formation process and composition. Comparative transcriptomic analyses between biofilm and planktonic *A. butzleri* cells would allow the identification of some other genes involved in biofilm formation and maturation. Similarly, they would also allow the identification of genes related to cellulose production if we compared producing and no producing strains on the basis of that observed in the Congo red binding assay. In this line, characterizing the composition of the biofilm matrix would be of great interest, as biofilms can be combated by targeting the extracellular polymeric compound. It would also allow establishing whether the detected cellulose is part of the biofilm matrix or not. Finally, a more in-depth characterization of the obtained *luxS* mutants will allow a further understanding of the QS-dependent processes in *A. butzleri* (i.e., pathogenicity), as well as studying the potential applicability of *Quorum Quenching* compounds as a strategy to control and prevent *A. butzleri* biofilms.

## 5. Conclusions

Our study sheds light on the role played by six genes (*flaA, flaB*, *fliS*, *luxS*, *pta* and *spoT*) in the biofilm formation capacity of *A. butzleri*, although future studies are necessary to achieve a satisfactory objective. In short, the *flaA*, *flaB*, *fliS* and *luxS* genes seem to play a positive role in the biofilm formation capacity of *A. butzleri*, while the *spoT* gene seems to play a negative one. Our results point to the genes *flaA*, *flaB*, *fliS*, *luxS* and *spoT* as interesting targets in the design and development of anti-biofilm strategies. Therefore, besides contributing to the general knowledge about biofilm in *Arcobacter*, this study sets the basis for future research on the prevention, control and eradication of biofilms produced by *A. butzleri*. Designing and developing strategies that facilitate the control of the biofilms formed by *Arcobacter* is of great importance in order to prevent the transmission of this potentially harmful bacteria, especially through the food chain. On the other hand, and according to our result, the Congo red binding assay is not a useful method to determine the biofilm production in *Arcobacter.*

## Figures and Tables

**Table 2:** Biofilm formation ability, motility and phenotype on CRA shown by the strains in this study.

Bacterial Strain	PS ^1^		Reinforced Glass		Stainless Steel (logUFC/cm^2^)	Motility (cm)	CRA ^4^
	BFI ^2^	Categ ^3^	BFI ^2^	Categ ^3^			
BER7	1.679 ± 0.609	S	0.462 ± 0.093	W	2.285 ± 0.292	2.833 ± 0.233	White
BER7Δ*pta*::Km	8.263 ± 3.108 *	S	0.732 ± 0.373	M	2.427 ± 0.341	0.000 *	White
CCUG 30485	5.140 ± 2.702	S	2.328 ± 0.574	S	2.877 ± 0.461	1.233 ± 0.145	Red
CCUG 30485Δ*flaAB*::Km	5.144 ± 1.981	S	0.965 ± 0.278	M	1.832 ± 0.113	0.000 *	Red
CCUG 30485Δ*fliS*::Km	2.571 ± 1.569	S	0.913 ± 0.223	M	3.444 ± 0.737	0.000 *	Red
CCUG 30485Δ*luxS*::Km	3.191 ± 0.421	S	1.008 ± 0.337	M	2.901 ± 0.494	1.233 ± 0.186	Red
CCUG 30485Δ*pta*::Km	5.498 ± 2.444	S	1.343 ± 0.276	S	3.000 ± 0.292	1.233 ± 0.133	Red
CCUG 30485Δ*spoT*::Km	8.502 ± 4.728	S	1.455 ± 0.449	S	3.858 ± 0.729	0.967 ± 0.145	Red
CH11	4.265 ± 0.772	S	1.179 ± 0.435 ^†^	S	4.233 ± 0.373	1.067 ± 0.033	White
CH11Δ*flaAB*::Km	2.822 ± 1.544	S	0.748 ± 0.205	M	3.433 ± 0.332	0.000 *	White
CH11Δ*fliS*::Km	3.171 ± 2.154	S	0.542 ± 0.187	W	4.322 ± 0.260	0.000 *	White
CH11Δ*luxS*::Km	2.650 ± 1.852	S	0.693 ± 0.200	W	3.934 ± 0.450	1.167 ± 0.067	White
CH11Δ*pta*::Km	4.997 ± 3.366	S	0.248 ± 0.096	N	4.367 ± 0.597	1.433 ± 0.203	White
CH11Δ*spoT*::Km	7.271 ± 4.163	S	0.331 ± 0.211	N	4.458 ± 0.456	1.200 ± 0.208	White
CZ6	0.000	N	0.851 ± 0.191	M	3.419 ± 0.213	2.533 ± 0.120	Red
CZ6Δ*fliS*::Km	0.007 ± 0.007	N	0.176 ± 0.102 *	N	4.032 ± 0.674	0.000 *	Red
CZ6Δ*luxS*::Km	0.000	N	0.379 ± 0.115	W	3.515 ± 0.224	2.800 ± 0.115	Red
CZ6Δ*pta*::Km	4.608 ± 3.979	S	0.312 ± 0.105	N	3.094 ± 0.321	2.267 ± 0.176	Red
CZ6Δ*spoT*::Km	4.852 ± 4.685	S	0.706 ± 0.211	M	4.700 ± 0.148 *	2.900 ± 0.058	Red
P8	17.319 ± 3.671 ^●^	S	3.825 ± 0.257	S	5.989 ± 0.140	2.600 ± 0.153	White
P8Δ*fliS*::Km	7.961 ± 1.448	S	1.752 ± 0.270 *	S	5.489 ± 0.222	0.000 *	White
P8Δ*luxS*::Km	13.706 ± 2.152	S	4.547 ± 0.752	S	5.944 ± 0.179	2.167 ± 0.088	White
P8Δ*pta*::Km	11.132 ± 1.304	S	3.516 ± 0.761	S	5.732 ± 0.109	2.300 ± 0.153	White

^1^ PS: Polystyrene. ^2^ BFI, Biofilm Formation Index. ^3^ Categ, categorization according to Naves et al., [88]: Strong (S), Moderate (M), Weak (W) and None (N). ^4^ CRA, phenotype shown on CRA plates. * Student’s *t*-based statistically significant (*p* < 0.05) differences obtained when comparing wild-type strains with their derivatives on each surface. ^●^ ANOVA-based statistically significant (*p* < 0.05) differences obtained when comparing the BFI values obtained on PS for each wild-type strain. ^†^ Student’s *t*-based statistically significant (*p* < 0.05) differences obtained when comparing biofilm formation on PS versus borosilicate.

## Data Availability

Not applicable.

## Supplementary Materials

The following supporting information can be downloaded at: www.mdpi.com/article/10.3390/microorganisms10071280/s1, Table S1: Primes used in this study, Figure S1: Growth curves of wild-type and mutant strains and their growth rates, Figure S2: Motility of the different wild and knockout strains, Figure S3: Colony growth pigmentation in CRA assay of the different wild and knockout strains.
