# Supplementary material for: Arcobacter butzleri Biofilms: Insights into the Genes Beneath Their Formation
Source: Microorganisms. 2022 Jun 23;10(7):1280. doi: 10.3390/microorganisms10071280 (PMC9324650; doi:10.3390/microorganisms10071280)
Supplement: Supplementary file 1 [file microorganisms-10-01280-s001.zip › microorganisms-1749967-supplementary.pdf]

Supplementary Materials

SUPPLEMENTARY TABLES

Table S1. Primes used in this study.

Target gene	Primer name	DNA sequence (5′ → 3′)	Annealing Temperature (°C)	Amplicon size (bp)	Reference
**Gene detection**					
flaA	*flaA*-F	CCAGCTGACATTTTAGCACCAC	50	145	[1]
	flaA-R	CTGCTGCAAGAACTGCAAAAGG			
flaB	*flaB*-F	TGCGTATGACCCTGCTTGTG	50	419	[1]
	flaB-R	CTGCTGCAAGAACTGCATTAGG			
fliS	*fliS*-F	AAAAGTGCAATACAAGAGGGTGA	50	114	[1]
	fliS-R	AGCAACATCTCCACCATCAAAA			
luxS	*luxS*-F	TATTAGATAGTTTTAGAGTTGA	50	256	[1]
	luxS-R	TAAAATCCAGTTCTACAACCCCAT			
pta	*pta*-F	AGATTTTTTGGTTGTATGATGGTAAGACT	50	431	[1]
	pta-R	GCAGCATCAGCTTGAAGCTCACCATCAAA			
spoT	*spoT*-F	TTGCCAATGAGCCGCAATTC	50	936	[1]
	spoT-R	AGCGGTGAACCTTACTGTGT			
**Mutant construction**					
flaAB	*flaAB*-F	CAGAAAATGGTGCTAAATTCTTAGG	53	2569	This study
	flaAB-R	TACCTTCGTAGAATTTACAATGTGTC			
	flaAB-F-*Bam*HI	AGGATCCGCTGGTGCAACTGCTGGTACAAC	60	5330	This study
	flaAB-R-*Bam*HI	AGGATCCTTTGCAATAGCCGTTCTACC			
fliS	*fliS*-F	TTCAACAGTCAACGGCAAGT	60	447	This study
	fliS-R	CCTGTTTCGTCTTTCCATGC			
luxS	*luxS*-F	TTTCTCTTTCGCAAACTCTCC	65	2392	This study
	luxS-R	TCGCATTTGGTGCTTGAAATCC			
pta	*pta*-F	AAGCGTAATCAGGCATAGTTTGG	65	2004	This study
	pta-R	TGTTCTTACGCTGGATTAATGC			
spot	*spoT*-F	TGGCTTTGAAGCAGACAAAA	65	1075	This study
	spoT-R	AATGGCTTCGTGACCATTTC			
**Kanamycin cassette amplification**					
aph(3′)-III	*km*F-*MunI*	CGCAATTGAGCGAACCATTTGAGGTGAT	58	1427	This study
	*km*R-*MunI*	CGCAATTGTTTAGACATCTAAATCTAGG			
	*km*F-*ClaI*	CGATCGATAGCGAACCATTTGAGGTGAT	56	1427	This study
	*km*R-*ClaI*	CGATCGATTTTAGACATCTAAATCTAGG			
	*km*F-*BmtI*	CGGCTAGCAGCGAACCATTTGAGGTGAT	60	1427	This study
	*km*R-*BmtI*	CGGCTAGCTTTAGACATCTAAATCTAGG			
	*km*F-*AflII*	CGCTTAAGAGCGAACCATTTGAGGTGAT	57	1427	This study
	*km*R-*AflII*	CGCTTAAGTTTAGACATCTAAATCTAGG			

Restriction sites are underlined.

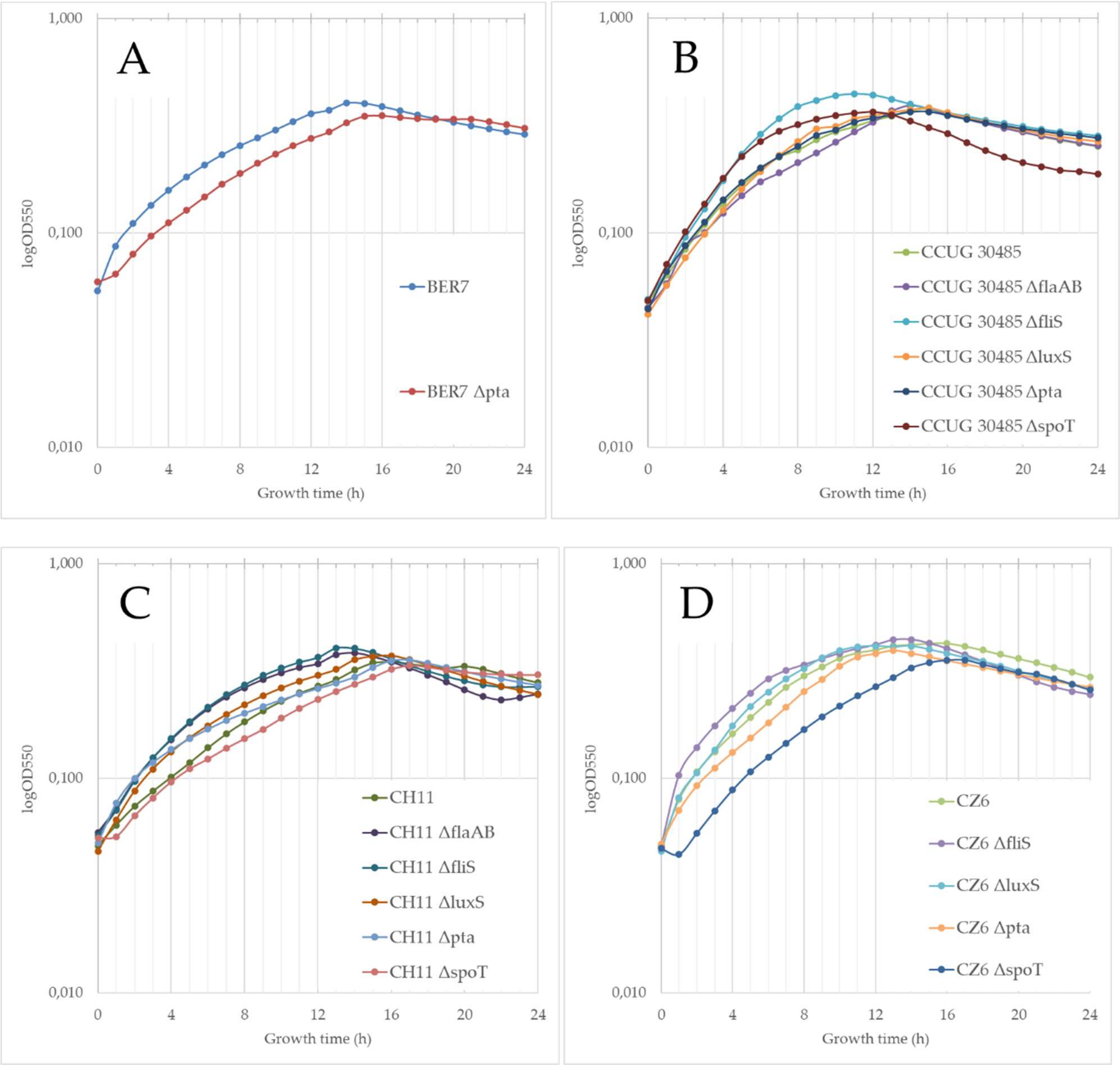

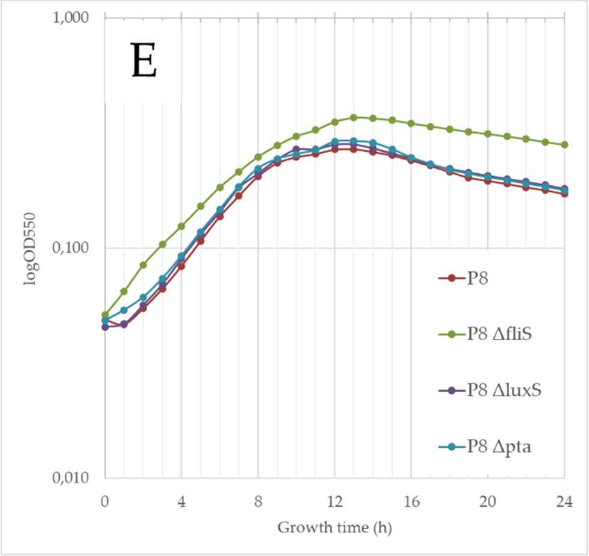

F

STRAINS	µ	SE
**BER7**	0.060	0.002
**BER7Δpta**	0.051	0.006
**CCUG 30485**	0.097	0.008
**CCUG 30485 ΔflaAB**	0.071	0.008
**CCUG 30485 ΔfliS**	0.102	0.007
**CCUG 30485 ΔluxS**	0.099	0.005
**CCUG 30485 Δpta**	0.081	0.004
**CCUG 30485 ΔspoT**	0.130	0.018
**CH11**	0.058	0.001
**CH11 ΔflaAB**	0.071	0.005
**CH11 ΔfliS**	0.054	0.005
**CH11 ΔluxS**	0.065	0.004
**CH11 Δpta**	0.050	0.003
**CH11 ΔspoT**	0.065	0.003
**CZ6**	0.077	0.006
**CZ6 ΔfliS**	0.081	0.001
**CZ6 ΔluxS**	0.094	0.006
**CZ6 Δpta**	0.071	0.002
**CZ6 ΔspoT**	0.072	0.013
**P8**	0.111	0.006
**P8 ΔfliS**	0.088	0.008
**P8 ΔluxS**	0.112	0.002
**P8 Δpta**	0.112	0.004

Figure S1. Growth curves of wild-type and mutant strains: (A) BER7 and derivatives; (B) CCUG 30485 and derivatives; (C) CH11 and derivatives; (D) CZ6 and derivatives; (E) P8 and derivatives; and (F) their growth rates. Growth rates (µ) are expressed as generations per hour. SE: Standard Error.

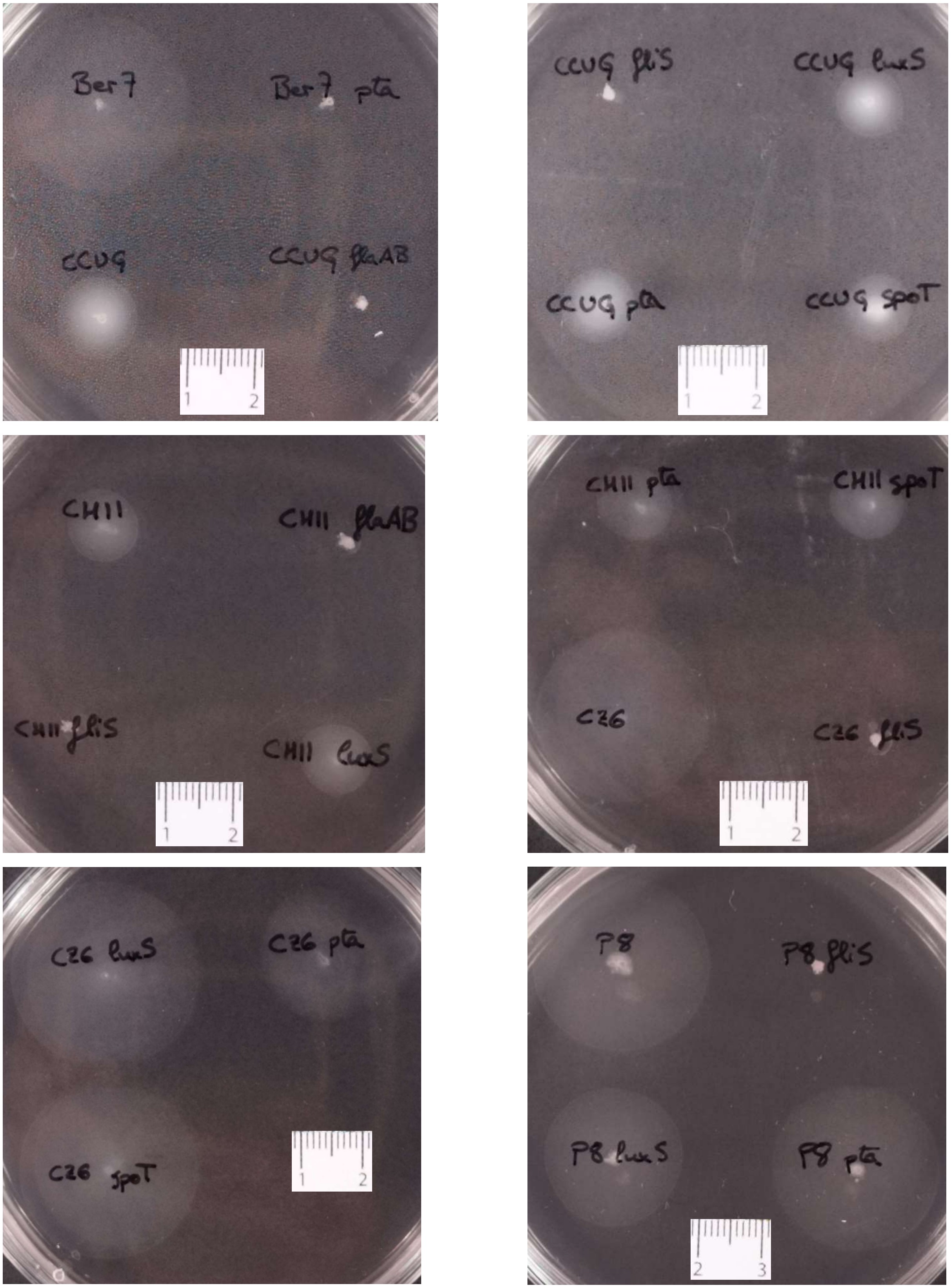

Figure S2. Images of a representative replicate obtained from the motility assay performed with studied *Arcobacter butzleri* wild and mutant strains. Studied strains are: BER7 (Ber7), BER7 Δ*pta* (Ber7 pta), CCUG 30485 (CCUG), CCUG 30485 Δ*flaAB* (CCUG flaAB), CCUG 30485 Δ*fliS* (CCUG fliS), CCUG 30485 Δ*luxS* (CCUG luxS), CCUG 30485 Δ*pta* (CCUG pta), CCUG 30485 Δ*spoT* (CCUG spoT), CH11 (CH11), CH11 Δ*flaAB* (CH11 flaAB), CH11 Δ*fliS* (CH11 fliS), CH11 Δ*luxS* (CH11 luxS), CH11 Δ*pta* (CH11 pta), CH11 Δ*spoT* (CH11 spoT), CZ6 (CZ6), CZ6 Δ*flis* (CZ6 fliS), CZ6 Δ*luxS* (CZ6 luxS), CZ6 Δ*pta* (CZ6 pta), CZ6 Δ*spoT* (CZ6 spoT), P8 (P8), P8 Δ*fliS* (P8 fliS), P8 Δ*luxS* (P8 luxS) and P8 Δ*pta* (P8 pta).

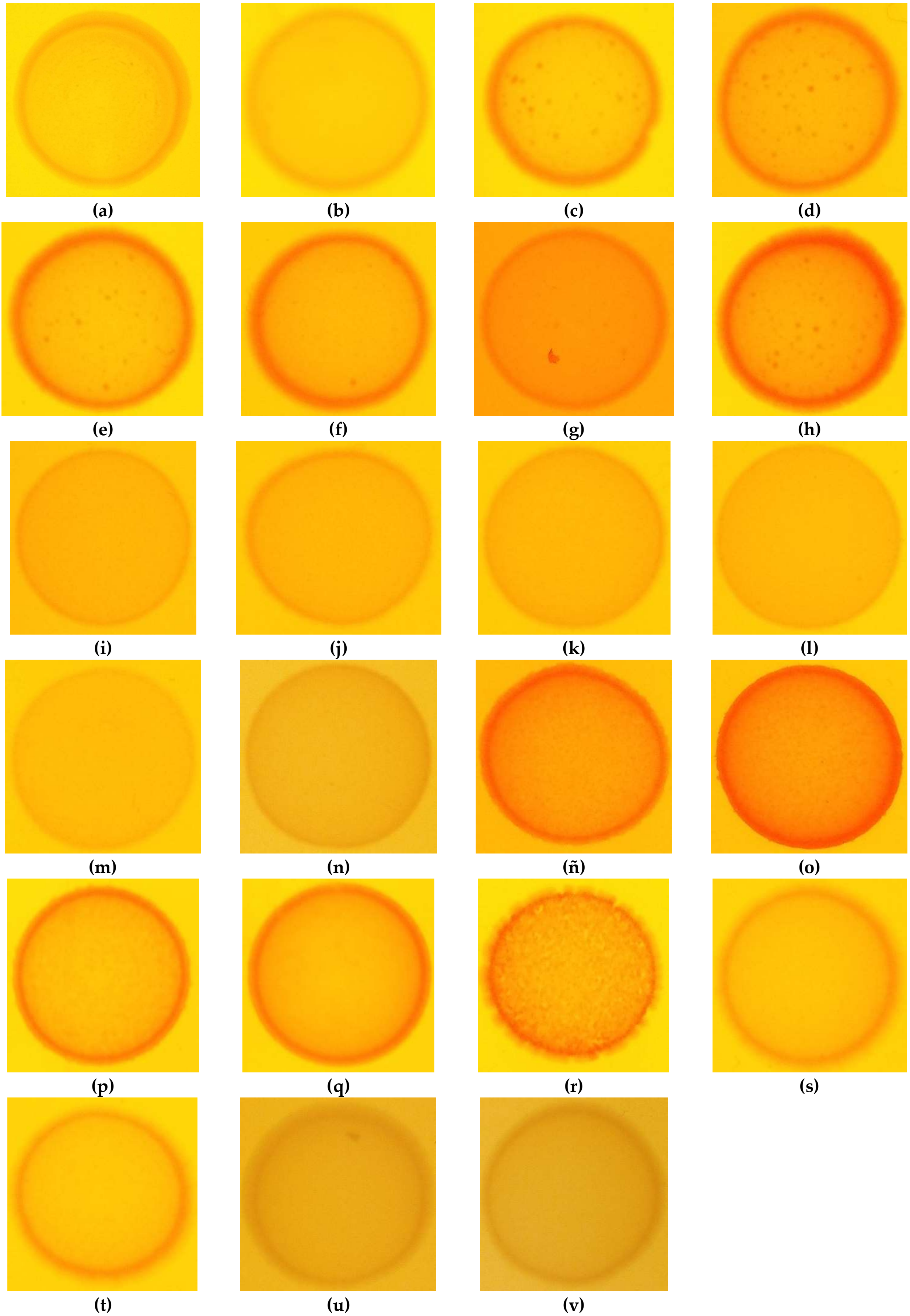

Figure S3. Colony growth pigmentation in CRA assay of the different wild and knockout strains: (a) AB-BER7; (b) AB--BER7 Δ*pta*::Km; (c) AB-CCUG 30485;(d) AB-CCUG 30485 Δ*flaAB*::Km; (e) AB-CCUG 30485 Δ*fliS*::Km; (f) AB-CCUG 30485 Δ*luxS*::Km; (g) AB-CCUG 30485 Δ*pta*::Km; (h) AB-CCUG 30485 Δ*spoT*::Km; (i) AB-CH11; (j) AB-CH11 Δ*flaAB*::Km; (k) AB-CH11 Δ*fliS*::Km; (l) AB-CH11 Δ*luxS*::Km; (m) AB-CH11 Δ*pta*::Km; (n) AB-CH11 Δ*spoT*::Km; (ñ) AB-CZ6; (o) AB-CZ6 Δ*fliS*::Km; (p) AB-CZ6 Δ*luxS*::Km; (q) AB-CZ6 Δ*pta*::Km; (r) AB-CZ6 Δ*spoT*::Km; (s) AB-P8; (t) AB-P8 Δ*fliS*::Km; (u) AB-P8 Δ*luxS*::Km; (v) AB-P8 Δ*pta*::Km.

SUPPLEMENTARY REFERENCES

1. Martinez-Malaxetxebarria, I.; Girbau, C.; Salazar-Sánchez, A.; Baztarrika, I.; Martínez-Ballesteros, I.; Laorden, L.; Alonso, R.; Fernández-Astorga, A. Genetic characterization and biofilm formation of potentially pathogenic foodborne *Arcobacter* isolates. *Int. J. Food Microbiol*. **2022**; DOI: 10.1016/j.ijfoodmicro.2022.109712.
